# Mechanisms of P-Glycoprotein Modulation by Semen Strychni Combined with Radix Paeoniae Alba

**DOI:** 10.1155/2017/1743870

**Published:** 2017-10-15

**Authors:** Li-Li Liu, Yong-Mei Guan, Xue-Ping Lu, Xin-Li Liang, Li-Hua Chen

**Affiliations:** ^1^Key Laboratory of Modern Preparation of TCM, Ministry of Education, Jiangxi University of TCM, 1688 Meiling Road, Nanchang, Jiangxi 330004, China; ^2^Science and Technology College of Jiangxi University of TCM, 819 Meiling Road, Nanchang, Jiangxi 330004, China

## Abstract

Semen Strychni has been extensively used as a Chinese herb, but its therapeutic window is narrowed by the strong toxicity of the compound, which limits its effectiveness. Radix Paeoniae Alba has been reported to reduce the toxic effects and increase the therapeutic effects of Semen Strychni, but the underlying mechanism remains unknown. This research aimed to explore the mechanism through which P-glycoprotein (P-gp) is modulated by Semen Strychni combined with Radix Paeoniae Alba in vitro. An MTT assay was used to study cytotoxicity in an MDCK-MDR1 cell model. Rh123 efflux and accumulation were measured to assess P-gp function. The expression levels of MDR1 mRNA and P-gp protein in MDCK-MDR1 cells were investigated. A P-gp ATPase activity assay kit was applied to detect the effect on P-gp ATPase activity. Semen Strychni combined with Radix Paeoniae Alba could induce P-gp-mediated drug transport by inhibiting brucine and strychnine transport in MDCK-MDR1 cells, enhancing the P-gp efflux function, upregulating the P-gp expression and MDR1 mRNA levels, and stimulating P-gp ATPase activity.

## 1. Introduction

Semen Strychni has been used in the treatment of joint pain, arthritis, and rheumatic diseases in China. Its main bioactive components,* Strychnos* total alkaloids, which comprise more than 70% of the alkaloids isolated from Semen Strychni, have been shown to exert antitumour, analgesic, anti-inflammatory, antioxidant, and antiangiogenesis effects as well as other practical effects [1]. Although it is widely used as a folk medicine, the use of Semen Strychni is limited by its narrow therapeutic window due to its strong excitatory effects on the central nervous system [2]. Some studies have demonstrated the appearance of persistent or transient neurovirulence in humans who excessively use Semen Strychni or its representative constituents, brucine, and strychnine. Therefore, it is very important to find a method to protect against the toxicity caused by Semen Strychni.

Protective herbal therapy is considered a reliable method that can reduce the toxicity of Semen Strychni and increase its use in clinical practice. Radix Paeoniae Alba has been reported to have wide-ranging pharmacological activities on the nervous system [3] and has been combined with Semen Strychni in the clinic and in the prescription of Chinese patent drugs, such as Gu Ci tablets, Shu Luo Yang Gan pills, and Qi Wei Tang. Radix Paeoniae Alba reportedly decreases the toxic effects and increases the therapeutic effects of Semen Strychni [4–6]. Additionally, Radix Paeoniae Alba and its active ingredients have shown protective effects in previous cytotoxicology experiments [7, 8].

P-gp is an energy-dependent transporter that relies on the energy released by ATP hydrolysis to transport the substrate to the extracellular domain. It can affect drug absorption in the intestinal tract, drug distribution to the brain, and drug elimination by the liver and kidneys [9]. Researchers have identified several Chinese herbal medicinal ingredients that can mediate P-gp efflux, including tetrandrine [10], dauricine [11], and quercetin [12]. Brucine and strychnine have been shown to be potential P-gp substrates [13]. Some research has indicated that P-gp takes part in the transport of brucine at the blood-brain barrier (BBB) [14]. As a result, the induction of P-gp activity can promote the removal of toxic drugs from the cell and meanwhile may cause potential herb-herb interactions. This is especially concerning for drugs with a narrow therapeutic window such as Semen Strychni. The clinical application of Semen Strychni is limited mainly by its nephrotoxicity and neurotoxicity. Moreover, paeoniflorin can reportedly cross the BBB [15]. Radix Paeoniae Alba extract has been reported to activate the P-gp efflux function [16]. Therefore, we speculated that the mechanism underlying the interaction between Radix Paeoniae Alba and Semen Strychni might be related to P-gp modulation. In this study, we focused on how the combination of Radix Paeoniae Alba and Semen Strychni affects P-gp. P-gp can be modulated by effects on its expression and function, drug binding sites, and ATPase activity as well as by changes in cell membrane fluidity.

As far as we know, there is no report regarding the mechanism underlying P-gp modulation by Semen Strychni combined with Paeoniae Radix Alba. So in the manuscript, we focused on the mechanism of P-gp modulation by Semen Strychni combined with Radix Paeoniae Alba in vitro. We evaluated cytotoxicity in MDCK-MDR1 cells, which stably express a high level of P-gp, using an MTT assay. The function of P-gp was estimated by determining the efflux and accumulation of Rh123. The expression level of P-gp and MDR1 mRNA in MDCK-MDR1 cells was measured by Western blotting analysis and RT-PCR. A P-gp ATPase activity assay kit was used to explore the effect on P-gp ATPase activity. The results of this research could be used not only to improve the clinical use of Semen Strychni but also to facilitate the analysis of protective herbal therapies. Furthermore, we hope that the results will provide information for the application of toxic Chinese medicinal materials.

## 2. Materials and Methods

### 2.1. Reagents

The RNA extraction reagent TRIzol was obtained from Ambion (USA). The PrimeScript RT reagent kit and Pgp-Glo™ assay system were purchased from Promega (USA). The PowerSYBR Green PCR Mix was obtained from Life Technologies, Inc. (USA).

### 2.2. Preparation of RPAE and SSE

We obtained* Radix Paeoniae Alba* extract (RPAE, containing 57.18% paeoniflorin, as determined by HPLC) as reported in the literature [17]. We also obtained* Semen Strychni* extract (SSE, containing 17.70% brucine and 35.95% strychnine, as determined by HPLC) as reported in the literature [18].

### 2.3. Cells

MDCK-MDR1 cells were bought from the Zhongya Biological Gene Institute (China) and cultured in DMEM containing 100 U/mL penicillin, 1% NEAA, 100 *μ*g/mL streptomycin, and 10% FBS at 37°C in an atmosphere of 5% CO_2_ at 95% relative humidity [19, 20]. The cells were passaged using a trypsin-EDTA solution every three to four days when they had grown to 80%–90% confluence.

### 2.4. MTT Assay

The cells (5 × 10^4^ cells/well) were seeded into a 96-well culture plate and cultured for 24 h. Fresh medium containing different test drugs was added to the relevant wells. After 4 h, the culture medium was removed, and the cells were washed twice with HBSS. Next, 20 *μ*L of MTT was put into each well and removed after 4 h. Then, add 200 *μ*L of DMSO to every well. The plate was incubated with shaking for 10 minutes, and the absorbance at 490 nm was read using a microplate reader (BioTek Elx800, USA). Wells with no cells were used as blank control wells, and wells which contained cells without the test drug were used as negative control wells.

### 2.5. Transport Experiment

Cells (1 × 10^5^ cells/cm^2^) were seeded onto polycarbonate filter membranes in a 12-well plate. The cell monolayers could be used for transport studies after the cells were allowed to grow for five to seven days. The transepithelial electrical resistance (TEER) of each MDCK-MDR1 cell monolayer was measured and exceeded 500 Ω/cm^2^. The cell monolayers were washed twice with HBSS and then incubated for 30 min. The HBSS was removed, and test drug solutions were added to either the apical (AP, 0.5 mL) or basolateral side (BL, 1.5 mL). Samples were taken from the receiving side after 2 h incubation. The samples were diluted with the same volume of methanol, mixed, and centrifuged at 16000 r/min at 4°C for 20 min to precipitate proteins. Brucine and strychnine were determined by LC-MS/MS.

### 2.6. LC-MS/MS Analysis

Brucine and strychnine in the samples were analysed by LC-MS/MS using an AB Triple Quad 4500 system (AB SCIEX). Brucine, strychnine, and the internal standard matrine were separated on a Gemini C_18_ column (150 mm × 4.6 mm ID, Phenomenex, USA) using acetonitrile-water containing 10 mmol/L ammonium acetate (30 : 70) as the mobile phase. An electrospray ionization (ESI) source was used and operated in the positive-ion mode. Brucine, strychnine, and matrine were detected at *m*/*z* 395.3 → 324.4, *m*/*z* 335.2 → 184.2, and *m*/*z* 249.3 → 148.3, respectively. Linear calibration curves were obtained for brucine and strychnine in the concentration range of 1.02–102.20 ng/mL. The lower limits of quantitation for brucine and strychnine, defined as a signal-noise ratio of 10, were 1.02 ng/mL and 1.20 ng/mL, respectively. The extraction recoveries were 95.75%–102.54% and 95.82%–104.64%, respectively. The precision, accuracy, and stability of the analytes met the requirements. The results showed that the method was effective and convenient for the detection of strychnine and brucine in the transport samples.

### 2.7. Flow Cytometric Analysis of the Intracellular Accumulation of Rh123

For the flow cytometric analysis, 1 mL aliquots of the cell suspension (1 × 10^6^ cells/mL) were added in Eppendorf (EP) tubes, and the test drug solutions were put into the tubes. The mixtures were incubated for 1 h at 37°C. 1 mL of Rh123 (10 *μ*M) was added and the mixtures were incubated for 1 h at 37°C and then resuspended in 0.5 mL of HBSS. The samples were detected by flow cytometry [21]. Verapamil is a well-known P-gp inhibitor as a positive control medicine.

### 2.8. qRT-PCR Analysis of MDR1 mRNA Expression

The cells (5 × 10^4^ cells/well) were seeded into a 12-well culture plate and then cultured for five days. The cells were incubated with test drugs for 2 h and washed twice with HBSS. Total RNA was extracted using TRIzol on the basis of the manufacturer's specifications. First-strand cDNA synthesis and amplification were performed on the basis of the Prime Script RT reagent kit protocol. qRT-PCR was performed as described in the Power SYBR Green PCR Mix protocol with a real-time fluorescent quantitative PCR platform (Prism 7500, ABI, USA). The following primers (Sangon, China) were used: MDR1 (F) 5′-TGG GGC TGG ACT TCC TCT CAT GAT GC-3′, MDR1 (R) 5′-GCA GCA ACC AGC ACC CCA GCA CCA AT-3′, RpIP1v (F) 5′-CCC TCA TTC TGC ACG ACG AT-3′, and RpIP1v (R) 5′-GGC TCA ACA TTT ACA CCG GC-3′. The RpIP1v mRNA levels were used for normalization. The fold-change in gene expression was calculated using the 2^−ΔΔCT^ method.

### 2.9. Western Blotting Analysis of the Effect of SSE Combined with RPAE on P-gp Expression

Cells (5 × 10^4^ cells/plate) were seeded into 10 cm culture plates and cultured for five days. The cells were treated with the test drug solutions for 24 h and then washed twice with HBSS. The cells were lysed with RIPA Lysis Buffer containing protease inhibitors and phosphatase inhibitors on ice on the basis of the manufacturer's specifications. The protein concentration of the samples was determined according to the BCA Protein Assay Reagent Kit protocol, and equal amounts of protein were analysed. LDS Sample Buffer (NuPAGE®, Life Technologies Inc., USA) was added to the protein samples, and the samples were incubated for 10 min in a 70°C waterbath. The proteins were fractionated on 10% Bis-Tris Mini Gels (NuPAGE, Life Technologies Inc., USA) by electrophoresis (200 V, 50 min) and transferred (200 mA, 2 h) onto PVDF transfer membranes. The membranes were blocked with 5% BSA for 2 h and washed three times with TBST. Afterward, the membranes were incubated with the relevant primary antibodies (anti-P-glycoprotein antibody F4, Sigma, USA) overnight at 4°C. The membranes were then incubated with the appropriate secondary antibodies (goat anti-mouse IgG, HRP, Comwin, China) for 1 h. The protein signals were then detected by enhanced chemiluminescence (ECL). GAPDH (Sigma, USA) was used as the loading control.

### 2.10. P-gp ATPase Activity Assay

P-gp ATPase activity was measured using Pgp-Glo Assay Systems which provided the necessary reagents for performing luminescent P-glycoprotein (P-gp) ATPase assays [21–23]. P-gp is an energy-dependent protein, which relies on the energy released by ATP hydrolysis to transport the substrate outside the cell. However, ATP enzymes need to be activated during the hydrolysis of ATP. So compounds that interact with P-gp can be identified as stimulators or inhibitors of its ATPase activity.

The Pgp-Glo Assay detects the effects of compounds on recombinant human P-gp in a cell membrane fraction. The assay relies on the ATP dependence of the light-generating reaction of firefly luciferase. ATP is first incubated with P-gp; then the P-gp ATPase reaction is stopped, and the remaining unmetabolized ATP is detected as a luciferase-generated luminescent signal. Pgp-dependent decreases in luminescence reflect ATP consumption by P-gp; thus, the greater the decrease in signal, the higher the P-gp activity. Accordingly, samples containing compounds that stimulate the P-gp ATPase will have significantly lower signals than untreated samples.

Pgp-Glo Assay System included Pgp-Glo assay buffer, ATP detection substrate (lyophilized), recombinant human P-gp membranes, MgATP, ATP detection buffer, Na_3_VO_4_, and verapamil. The Pgp-Glo assays were performed in two steps. The first step was the P-gp reaction. In this step, a recombinant human P-gp membrane fraction was incubated in Pgp-Glo assay buffer with MgATP (5 mM) for 40 min at 37°C. Untreated (NT) and Na_3_VO_4_ (100 *μ*M) treated control samples were included except for verapamil (200 *μ*M) treated samples (positive control). When an ATP standard curve was used, the ATP standards were added to the plate at the end of this step. The second step was the ATP detection reaction. ATP detection reagent was added to the P-gp reaction described above and incubated for 20 min at 37°C. The samples were measured by a Varioskan Flash reader (Thermo Scientific). The statistics of the luminescent signals were on the basis of the manufacturer's specifications, and the influence of the test drug on* P-gp *ATPase activity was then estimated.

### 2.11. Statistical Analysis

The data were analysed by SPSS 19.0. The statistical difference between two groups was measured by* t*-test.

## 3. Results

### 3.1. MTT Assay

**Figure 1:**
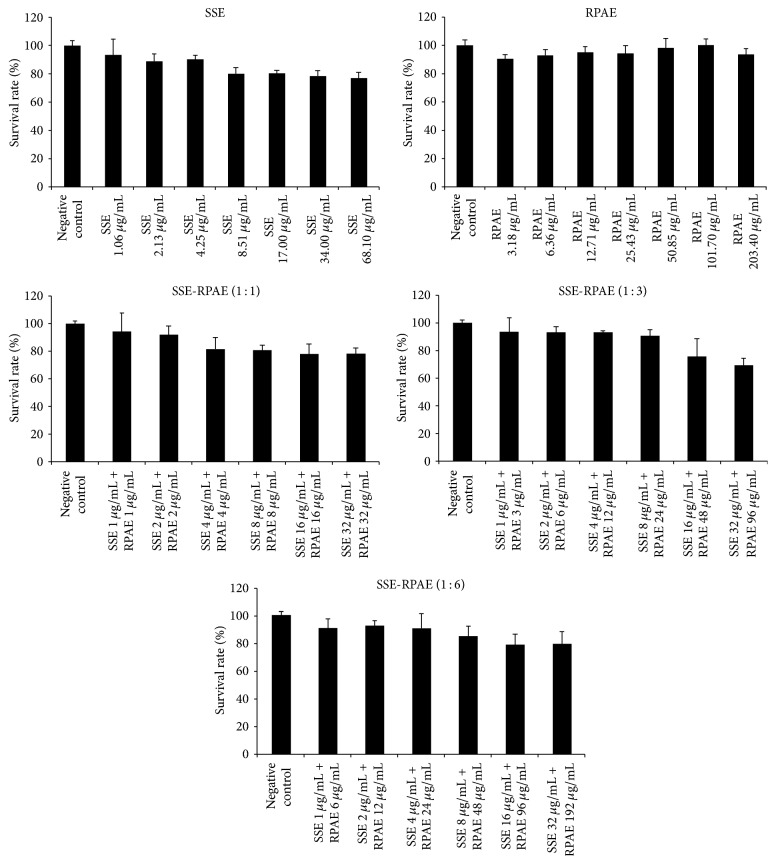
Toxicity of SSE, RPAE, and SSE and RPAE combined in MDCK-MDR1 cells.

The MTT assay results shown in Figure 1 indicated that SSE was nontoxic below the concentration of 1.06 *μ*g/mL and that RPAE was nontoxic in the range of 0–203.40 *μ*g/mL. When SSE and RPAE were combined, the nontoxic concentration range of SSE increased significantly. In particular, when the ratio of SSE to RPAE was 1 : 3, the nontoxic concentration range of SSE was 0–8.00 *μ*g/mL.

### 3.2. Effect of RPAE on Brucine and Strychnine Transport in MDCK-MDR1 Cells

As shown in Tables 1 and 2, the Papp_(AP-BL)_ values of brucine and strychnine in the SSE + RPAE groups were lower than those in the SSE group significantly, whereas the Papp_(BL-AP)_ values were higher. Moreover, the ER values in the SSE + RPAE groups were higher than those in the SSE group. As the RPAE concentration increased, the ER value increased. These results showed that the combination of SSE and RPAE inhibited the absorption of brucine and strychnine. In addition, the ER values were significantly lower in the groups treated with verapamil than in the groups without verapamil, indicating that P-gp was participated in the transport of brucine and strychnine.

### 3.3. Effect of RPAE and/or SSE on the Accumulation of Rh123

**Figure 2:**
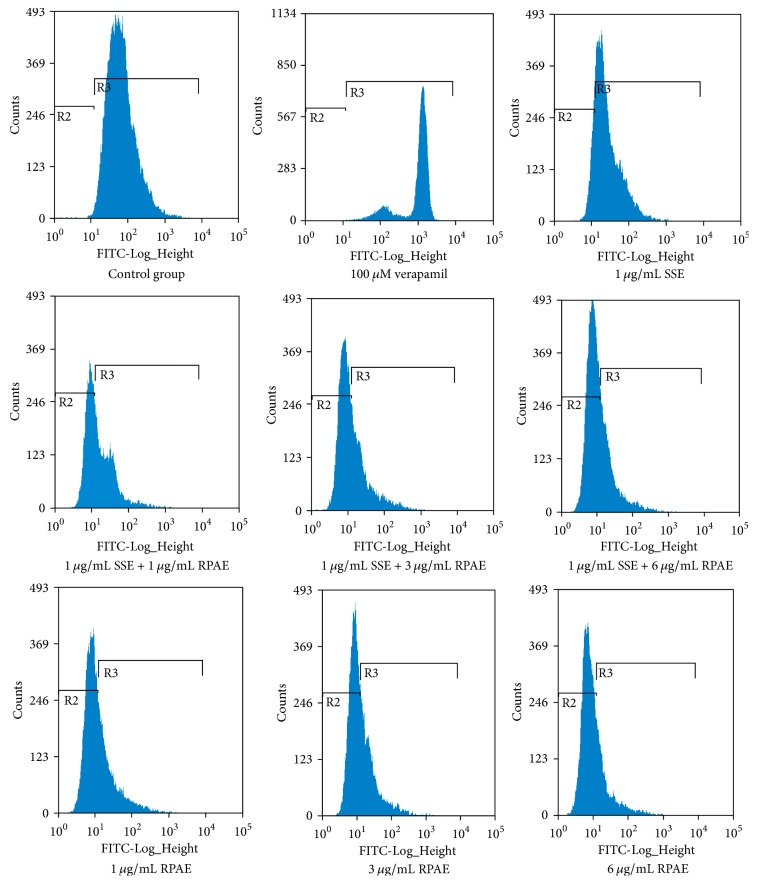
Effect of the combination of Semen Strychni and Radix Paeoniae Alba on P-gp function.

The accumulation of Rh123 was determined by flow cytometry. As shown in Table 3 and Figure 2, RPAE and SSE both decreased the intracellular accumulation of Rh123 compared with the control group. The intracellular accumulation of Rh123 in the SSE + RPAE groups was significantly lower than that in the SSE group, indicating that RPAE could activate the efflux function of P-gp.

### 3.4. Effect of RPAE and/or SSE on the Relative Expression Level of MDR1 mRNA

**Figure 3:**
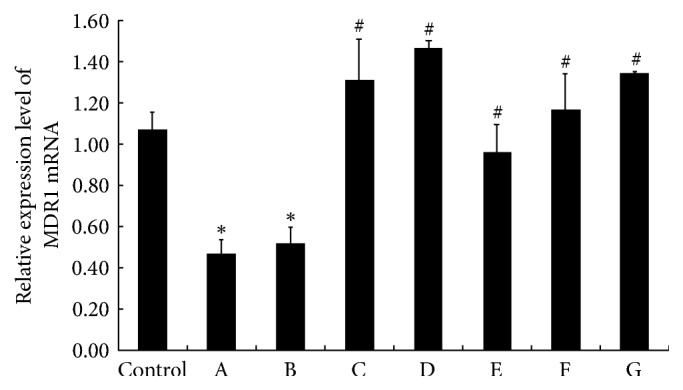
The effect of RPAE and/or SSE on the relative expression level of MDR1 mRNA was measured by qRT-PCR (*n* = 3). In the figure, A is the SSE (1 *μ*g/mL) group, B is the SSE (1 *μ*g/mL) + RPAE (1 *μ*g/mL) group, C is the SSE (1 *μ*g/mL) + RPAE (3 *μ*g/mL) group, D is the SSE (1 *μ*g/mL) + RPAE (6 *μ*g/mL) group, E is the RPAE (1 *μ*g/mL) group, F is the RPAE (3 *μ*g/mL) group, and G is the RPAE (6 *μ*g/mL) group. ^*∗*^*P* < 0.05 versus the control group. ^#^*P* < 0.05 versus the SSE (1 *μ*g/mL) group.

As shown in Figure 3, in contrast to the control group, the relative expression level of MDR1 mRNA was decreased significantly in the SSE group but increased in RPAE groups. The relative expression levels of MDR1 mRNA in the SSE and RPAE combination groups were higher than that in the SSE group. The relative expression levels of MDR1 mRNA in the SSE (1 *μ*g/mL) + RPAE (3 *μ*g/mL) and SSE (1 *μ*g/mL) + RPAE (6 *μ*g/mL) groups were higher than that in the control group.

### 3.5. Effect of RPAE and/or SSE on P-gp Expression

**Figure 4:**
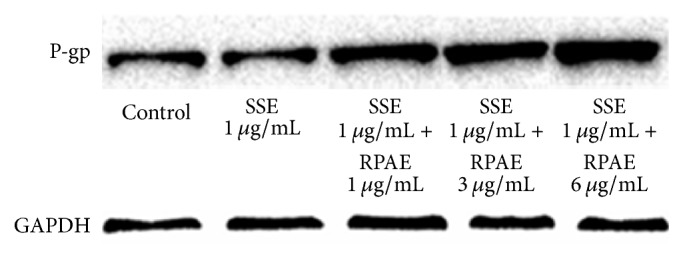
The effect of RPAE and SSE on the relative expression level of P-gp was measured by Western blot.

As shown in Figure 4 and Table 4, in contrast to the control group, P-gp expression significantly increased in the SSE and RPAE combination groups and significantly decreased in the SSE group.

### 3.6. Effect of RPAE and/or SSE on P-gp ATPase Activity

**Figure 5:**
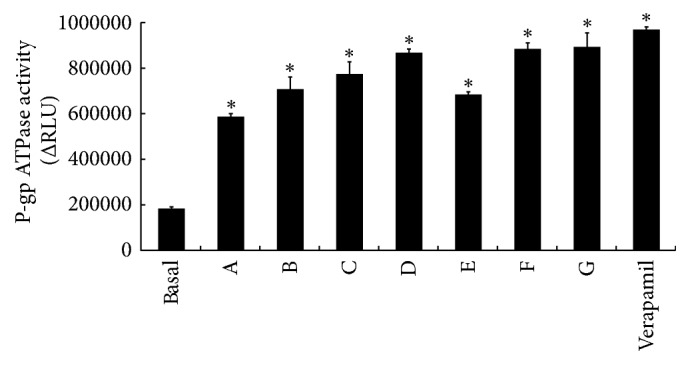
Effect of RPAE and/or SSE on P-gp ATPase activity (*n* = 3). In the figure, A is the SSE (1 *μ*g/mL) group, B is the SSE (1 *μ*g/mL) + RPAE (1 *μ*g/mL) group, C is the SSE (1 *μ*g/mL) + RPAE (3 *μ*g/mL) group, D is the SSE (1 *μ*g/mL) + RPAE (6 *μ*g/mL) group, E is the RPAE (1 *μ*g/mL) group, F is the RPAE (3 *μ*g/mL) group, and G is the RPAE (6 *μ*g/mL) group. ^*∗*^*P* < 0.05 versus the control group.

As shown in Figure 5, the ΔRLU of all groups was greater than ΔRLU_basal_, demonstrating that all of the test compounds are stimulators of P-gp ATPase activity. Because the ΔRLU in the RPAE groups was greater than that in the SSE group, the affinity of RPAE for P-gp was greater than that of SSE.

## 4. Discussion

Semen Strychni can effectively be used to treat inflammation and pain, but its use is limited by its toxic effects. Some studies have reported that Radix Paeoniae Alba can reduce the toxic effects and increase the therapeutic effects of Semen Strychni [4–6]. In the research, the mechanism of P-gp modulation by Semen Strychni combined with Radix Paeoniae Alba was investigated. First, the cytotoxic effects of SSE, RPAE, and the combination of SSE and RPAE on MDCK-MDR1 cells were estimated by an MTT assay with verapamil as the positive control. We found that the nontoxic concentration range of SSE increased significantly when combined with RPAE.

To investigate the underlying mechanism, the effect of RPAE on brucine and strychnine transport in MDCK-MDR1 cells was investigated, and the results showed that RPAE could inhibit brucine and strychnine transport in MDCK-MDR1 cells which might explain how RPAE reduces the toxicity of SSE to the central nervous system. Our results are consistent with those of previous studies demonstrating that P-gp prevented brucine from passing through the BBB in an in vitro model [10]. Rh123 efflux and accumulation were detected in MDCK-MDR1 cells by measuring Rh123-associated MFI. As far as we know, this report provides the first flow cytometry results demonstrating that the combination of SSE and RPAE induces a decrease in the intracellular accumulation and increases the efflux of Rh123 in a proportion-dependent manner. We showed that one of the mechanisms through which Radix Paeoniae Alba reduces the toxicity of Semen Strychni might be related to the inhibition of the efflux function of P-gp. To further investigate the regulatory effects of SSE + RPAE on P-gp, the expression of MDR1 mRNA and P-gp protein in MDCK-MDR1 cells was examined using Western blotting and RT-PCR analyses. The expression levels of MDR1 mRNA and P-gp in MDCK-MDR1 cells were significantly increased in the SSE + RPAE groups. These results indicated that the combination of SSE and RPAE could upregulate the expression of P-gp. Therefore, we speculated that SSE combined with RPAE could activate P-gp to increase the efflux of toxic components and that RPAE could induce the expression of P-gp.

Moreover, both SSE and RPAE could stimulate P-gp ATPase activity, demonstrating that SSE and RPAE directly interact with P-gp. The effect of RPAE on the activity of P-gp ATPase was stronger than that of SSE. The effect of RPAE and SSE combined on P-gp ATPase activity was also greater than that of SSE. Thus, Semen Strychni combined with Radix Paeoniae Alba could induce P-gp-mediated drug transport by upregulating the MDR1 mRNA expression level, increasing the P-gp expression level and stimulating P-gp ATPase activity.

In addition, membrane stability is closely related to drug transport. Therefore, the effect of the combination of RPAE and SSE on membrane lipid fluidity was also studied. RPAE, SSE, and their combination had no significant effects on membrane lipid fluidity. This result indicated that the mechanism through which P-gp is regulated by Semen Strychni combined with Radix Paeoniae Alba might not be related to membrane lipid fluidity.

Finally, although P-gp is the most well-known active efflux transporter, there are many other multidrug transporters, such as members of the multidrug resistance protein family. Our study focused on P-gp, but future studies should be performed to investigate the other transporters.

## 5. Conclusions

In the current study, we focused on the mechanism through which P-gp was regulated by the combination of SSE with RPAE and found that Semen Strychni combined with Radix Paeoniae Alba could induce P-gp-mediated drug transport, likely by inhibiting brucine and strychnine transport in MDCK-MDR1 cells, enhancing the P-gp efflux function, upregulating the MDR1 mRNA and P-gp expression levels, and stimulating P-gp ATPase activity. This investigation can not only improve the clinical therapeutic use of Semen Strychni but also might facilitate the exploration of possible preventative methods to decrease the toxic effects of Semen Strychni through its combination with Radix Paeoniae Alba.

## Figures and Tables

**Table 1:** Effect of RPAE on brucine transport in MDCK-MDR1 cells ($\overset{-}{x}\pm SD$, *n* = 3).

Groups	Papp_(AP-BL)_ (×10^−6^ cm/s)	Papp_(BL-AP)_ (×10^−6^ cm/s)	ER
1 *μ*g/mL SSE	6.81 ± 1.76	36.72 ± 0.23	5.39 ± 0.13
1 *μ*g/mL SSE + 1 *μ*g/mL RPAE	5.97 ± 0.58	33.82 ± 0.67	5.66 ± 1.16
1 *μ*g/mL SSE + 3 *μ*g/mL RPAE	4.63 ± 1.59^*∗*^	34.65 ± 1.30	7.48 ± 0.82
1 *μ*g/mL SSE + 6 *μ*g/mL RPAE	4.46 ± 0.97^*∗*^	48.23 ± 1.66^*∗*^	10.81 ± 1.71
1 *μ*g/mL SSE + 100 *μ*M verapamil	24.98 ± 2.17^#^	28.78 ± 2.16^#^	1.15 ± 0.99

^*∗*^ *P* < 0.05 with contrast to the SSE 1 *μ*g/mL group; ^#^*P* < 0.05 with contrast to groups without verapamil.

**Table 2:** Effect of RPAE on strychnine transport in MDCK-MDR1 cells ($\overset{-}{x}\pm SD$, *n* = 3).

Groups	Papp_(AP-BL)_ (×10^−6^ cm/s)	Papp_(BL-AP)_ (×10^−6^ cm/s)	ER
1 *μ*g/mL SSE	28.33 ± 2.67	39.39 ± 11.75	1.39 ± 4.40
1 *μ*g/mL SSE + 1 *μ*g/mL RPAE	24.49 ± 1.63	30.73 ± 0.64	1.25 ± 0.39
1 *μ*g/mL SSE + 3 *μ*g/mL RPAE	22.29 ± 8.19	37.39 ± 0.95	1.68 ± 0.12
1 *μ*g/mL SSE + 6 *μ*g/mL RPAE	19.64 ± 2.33^*∗*^	41.92 ± 1.53	2.13 ± 0.67
1 *μ*g/mL SSE + 100 *μ*M verapamil	44.19 ± 4.04^#^	39.57 ± 1.53	0.90 ± 0.38

^*∗*^ *P* < 0.05 with contrast to the SSE 1 *μ*g/mL group; ^#^*P* < 0.05 with contrast to groups without verapamil.

**Table 3:** Accumulation of Rh123 in MDCK-MDR1 cells ($\overset{-}{x}\pm SD$, *n* = 3).

Group	Fluorescence value
Control	111.95 ± 1.04
100 *μ*M verapamil	1113.13 ± 56.80^#^
1 *μ*g/mL SSE	46.14 ± 1.05^#^
1 *μ*g/mL SSE + 1 *μ*g/mL RPAE	30.37 ± 1.62^#*∗*^
1 *μ*g/mL SSE + 3 *μ*g/mL RPAE	26.07 ± 1.05^#*∗*^
1 *μ*g/mL SSE + 6 *μ*g/mL RPAE	24.77 ± 4.82^#*∗*^
1 *μ*g/mL RPAE	26.94 ± 0.84^#*∗*^
3 *μ*g/mL RPAE	25.70 ± 0.38^#*∗*^
6 *μ*g/mL RPAE	19.66 ± 0.30^#*∗*^

^#^ *P* < 0.05 with contrast to the control group; ^*∗*^*P* < 0.05 with contrast to the SSE 1 *μ*g/mL group.

**Table 4:** The effect of RPAE and SSE on the relative expression level of P-gp was measured by Western blot.

Relative protein quantification	Control	A	B	C	D
P-gp	1.00	0.88	1.53	2.17	2.79
GAPDH	1.00	1.00	1.00	1.00	1.00

In Table 4, A is the SSE (1 *μ*g/mL) group, B is the SSE (1 *μ*g/mL) + RPAE (1 *μ*g/mL) group, C is the SSE (1 *μ*g/mL) + RPAE (3 *μ*g/mL) group, and D is the SSE (1 *μ*g/mL) + RPAE (6 *μ*g/mL) group.
